# Indole-Based Hydrazones Containing A Sulfonamide Moiety as Selective Inhibitors of Tumor-Associated Human Carbonic Anhydrase Isoforms IX and XII

**DOI:** 10.3390/ijms20092354

**Published:** 2019-05-12

**Authors:** Kübra Demir-Yazıcı, Silvia Bua, Nurgül Mutlu Akgüneş, Atilla Akdemir, Claudiu T. Supuran, Özlen Güzel-Akdemir

**Affiliations:** 1 Department of Pharmaceutical Chemistry, Faculty of Pharmacy, Istanbul University, 34116 Istanbul, Turkey; kubra.demir@istanbul.edu.tr (K.D.-Y.); akgunes.nurgul@gmail.com (N.M.A.); 2 Department of NEUROFARBA, Sezione di Scienze Farmaceutiche Universita degli Studi di Firenze, Sesto Fiorentino, 50019 Florence, Italy; silvia.bua@unifi.it (S.B.); claudiu.supuran@unifi.it (C.T.S.); 3 Computer-Aided Drug Discovery Laboratory, Department of Pharmacology, Faculty of Pharmacy, Bezmialem Vakif University, 34093 Istanbul, Turkey; aakdemir@bezmialem.edu.tr

**Keywords:** carbonic anhydrase, sulfonamides, hydrazones, enzyme inhibition, molecular modeling

## Abstract

Novel sulfonamidoindole-based hydrazones with a 2-(hydrazinocarbonyl)-3-phenyl-1*H*-indole-5-sulfonamide scaffold were synthesized and tested in enzyme inhibition assays against the tumor-associated carbonic anhydrase isoforms, hCA IX and XII, and the off-targets, hCA I and II. The compounds showed selectivity against hCA IX and XII over hCA I and II. Six compounds showed ***K*_I_** values lower than 10 nM against hCA IX or XII. Molecular modeling studies were performed to suggest binding interactions between the ligand and the hCA active sites.

## 1. Introduction

Carbonic anhydrases (CA) are metalloenzymes that catalyze the reversible hydration of CO_2_ to HCO_3_^−^ and H^+^. This simple but important reaction is widespread amongst many if not all organisms [1]. Many of the six structurally different classes of CAs (i.e., α, β, γ, δ, ζ, and ηCAs) contain a Zinc ion (Zn^2+^) at the active site that is essential for catalysis [2]. CAs have several important physiological and pathological functions, such as respiration and transporting CO_2_/HCO_3_^−^ between metabolizing tissues and lungs, electrolyte secretion [3], pH regulation [4], and biosynthetic reactions such as gluconeogenesis, lipogenesis, ureagenesis [5] and carcinogenicity [6].

The human carbonic anhydrases (hCAs) are a member of the αCAs, which are divided into 16 different isozymes (hCA I-XVI) [7]. These enzymes share similar structure, especially in the active site, but show different catalytic activities and tissue distributions [8]. Cytosolic hCA isoforms I and II (hCA I and hCA II) are widespread in the human body and are targets of clinically used diuretics [9], antiglaucoma [10], drugs, and anticonvulsants [11]. In contrast, transmembrane isoforms, hCA IX and hCA XII, which have an active site on the extracellular part of the cell membrane, are located mainly on hypoxic tumor cells [12] and are validated drug targets for the design of anticancer agents specific for solid hypoxic tumors [13]. Proliferation and survival of tumor cells seem to be closely related to overexpression of both enzymes [14]. Thus, selective inhibition of hCA IX and hCA XII with well characterized CA inhibitors (CAIs) may result in new drugs in chemotherapy.

Sulfonamides, a main class of CAIs, show their effect by binding to the Zn^2+^ ion of the hCA active site. Many compounds with a sulfonamide moiety are in clinical use as CAIs or at the development process. Most of the sulfonamides act as strong CAIs for many hCAs—including isoforms I and II—causing a wide range of side effects. However, the design of new CAIs, which show selective inhibition for tumor-associated isoenzymes, hCA IX and XII, and weak affinity for hCA I and II, currently receives great attention in medicinal chemistry research [15,16,17]. Indole based sulfonamide derivatives were investigated during the last years and have been evaluated as selective inhibitors of several classes of carbonic anhydrases, including human/mammalian isoforms and pathogens [18,19]. Members of our group investigated 2-(hydrazinocarbonyl)-3-phenyl-1*H*-indole-5-sulfonamide for their interaction with 12 carbonic anhydrase isoforms in the search of compounds with good inhibitory activity against isozymes, such as CA I, II, VA, VB, VII, IX, and XII, among others [20]. Thus, we explore here the synthesis and structure–activity relationship (SAR) for the inhibition of four CA isoforms (hCA I, II, IX, and XII) with hydrazone derivatives of these compounds as putative CAIs.

## 2. Results

### 2.1. Chemistry

**Scheme 1:**
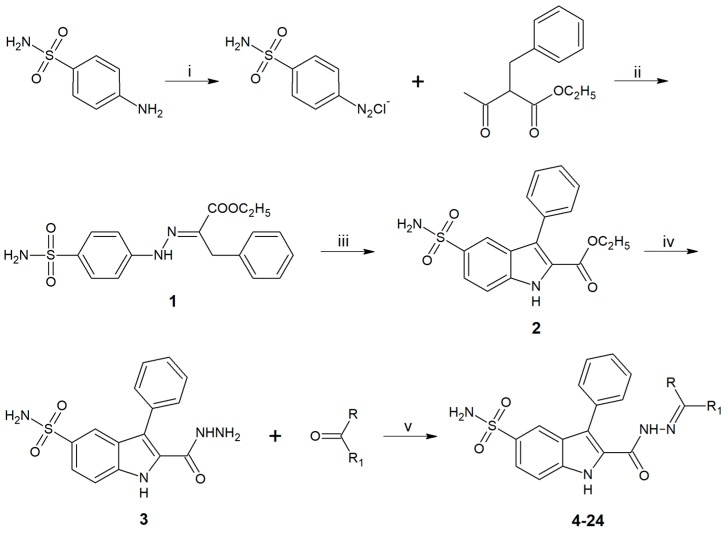
Reagents and conditions: (**i**) NaNO_2_, HCl, 0 °C; (**ii**) KOH, 0 °C; (**iii**) 37% HCl, reflux, 4 h; (**iv**) H_2_NNH_2_.H_2_O, EtOH, reflux, 6 h; (**v**) reflux, 6–12 h.

As was previously reported by our group, 3-phenyl-5-sulfonamido-1*H*-indole-2-carbohydrazide **1** was prepared from sulfanilamide as outlined in Scheme 1. After diazotization of the sulfanilamide, condensation of diazonium salt with ethyl 2-benzylacetoacetate **2** led to an intermediate, which was cyclized in acidic medium with formation of the ethyl ester derivative of **3**, which was converted to **3** by treatment with hydrazine [20]. Further treatment of **3** with an appropriate carbonyl compound (ketone or aldehyde) yielded hydrazone derivatives **4-24** (Scheme 1).

Newly synthesized hydrazones were characterized with melting points and spectral analyses. The IR spectra of compounds **4-24** showed NH stretching bands of the hydrazide-hydrazone group and sulfonamide moiety at the indole ring at 3423–3138 cm^−1^. The carbonyl functionalities of the new compounds were confirmed by strong C=O stretching bands observed in the 1643–1685 cm^−1^, while compound **3** showed a band of 1627 cm^−1^, as expected. Asymmetric and symmetric SO_2_ stretching vibrations of the sulfonamide group had absorption bands in the 1344–1311 cm^−1^ and 1176–1147 cm^−1^ [21,22].

The ^l^H-NMR spectrum of compounds displayed the N-H, the C_4_-H, the C_6_-H, and the C_7_-H protons of the indole ring at 12.39–12.14 ppm (for **9,** the second indole ring N-H proton was at 11.59 ppm) as a singlet and 8.85–7.15 ppm, 7.75–7.71, and 7.65–7.61 ppm, respectively. The N-H protons of the hydrazide structure exhibited the expected singlets at the 11.75–9.01 ppm [19]. The SO_2_NH_2_ protons had signals at 7.21–7.15 ppm. For compounds **4-11,** the azomethine protons resonated at 9.24–8.09 ppm except **10**. A broad resonance with 2H integration value at 8.21 and 8.24 ppm was assigned to the two azomethine protons of **10**. All the other protons were observed in the expected regions. In the ^13^C NMR spectrum of compounds, carbon atoms of the hydrazide carbonyl (CONH) and the hydrazone (C=N) groups were observed at in the order of 167.18–157.75 and 157.50–143.19 ppm.

### 2.2. Enzyme Inhibition Assays

**Figure 1:**
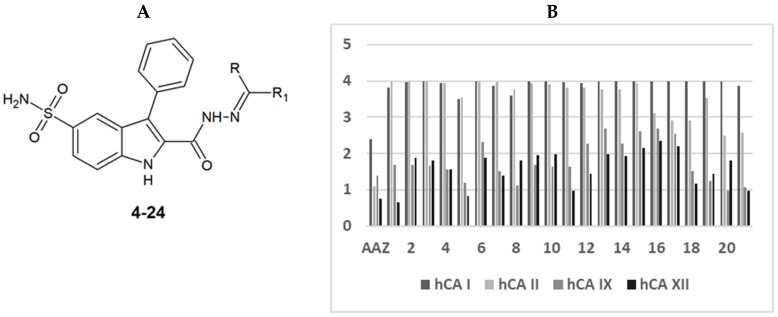
(**A**) New 2-(hydrazinocarbonyl)-3-phenyl-1*H*-indole-5-sulfonamide based hydrazone derivatives (substitutions are shown in Table 1). (**B**) Bar-graph showing the log *K*_I_ values of **AAZ** (acetazolamide) and the tested compounds.

All synthesized compounds were tested in enzyme inhibition assays against the widespread and cytosolic hCA I and II enzymes and the tumor-associated and transmembrane hCA IX and XII enzymes with extracellular catalytic domains (Table 1, Figure 1). The compounds showed selectivity for hCA IX and XII over hCA I and II (Table 1, Figure 1). For hCA IX, 14 of the tested compounds had ***K*_I_** values smaller than 100 nM, including one compound with a ***K*_I_** value of less than 10 nM (i.e., compound **23**). Of the 14 compounds, seven had a similar or lower ***K*_I_** value compared to acetazolamide (AAZ). For hCA XII, five of the tested compounds had a similar or lower ***K*_I_** value compared to acetazolamide. Compound **23** was selective for hCA IX with ~seven-fold selectivity over hCA XII and at least ~33-fold selectivity over the off-targets, hCA I and II. Additionally, this compound had a lower ***K*_I_** value compared to acetazolamide (AAZ).

Compound **17** had a cyclohexyl moiety, which was not very well tolerated (Table 1, Figure 1). Adding flexible aliphatic substituents to the cyclohexyl moiety increased the ***K*_I_** value of the ligand for hCA IX, while adding an aromatic phenyl or bulky *tert*-butyl substituent (compounds **21** and **22,** respectively) decreased the ***K*_I_** values (Table 1, Figure 1). A cyclopentyl instead of a cyclohexyl moiety was also not tolerated.

Compound **18**, the analog of compound **23** without a cationic nitrogen atom, showed a ~45-fold higher ***K*_I_** value compared to compound **23**. This may indicate that a cationic charge was favorable in the ligand binding interactions.

The compounds had, in general, lower ***K*_I_** values for hCA XII, with 18 compounds showing ***K*_I_** smaller than 100 nM, including five compounds with ***K*_I_** smaller than 10 nM. Compound **7** had the lowest measured ***K*_I_** value for hCA XII (***K*_I_**: 1.4 nM).

The 2-(hydrazinocarbonyl)-3-phenyl-1*H*-indole-5-sulfonamide was previously tested by members of our group against the tumor-associated hCA IX and XII and the off-targets, hCA I and II [20]. Compared to this previous compound, the newly synthesized hydrazon compounds had higher selectivity for hCA IX/XII compared to the off-targets.

### 2.3. Molecular Modeling Studies of hCA IX

**Figure 2:**
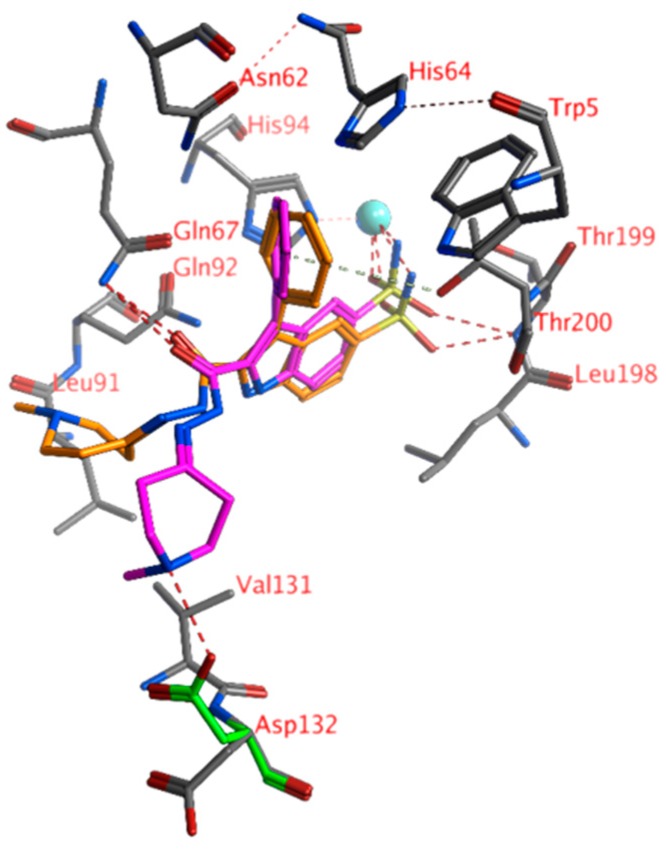
The docked pose of compound **23** (orange and purple) in the active site of hCA IX (3iai). Hydrogen bonds and interactions with the zinc ion are indicated in red dashed lines. H-arene interactions are indicated in yellow dashed lines. The zinc ion is indicated as a turquoise sphere. The Asp132 rotamer that was able to form a hydrogen bond with the ligand is indicated in green sticks.

Compound **23** displayed the lowest ***K*_I_** value for hCA IX of 9.2 nM. The sulfonamide moiety formed direct interactions with the Zn^2+^ ion (tetrahedral orientation) and the backbone of Thr199 (Figure 2; **23** pose in orange). The indole group of the ligand was situated between Gln92 and Leu198. The side chain hydroxyl group of Thr200 pointed with its hydrogen atom towards the centroid of the ligand’s phenyl group to interact with the π-cloud. A hydrogen bond was formed between the ligand’s carbonyl group and the side chain of Gln67. The cationic aliphatic ring of the ligand was located between Gln67, Leu91, and Val131. The latter cationic group did not form any interactions with the active site residues. Detailed investigation of the ligand’s docked pose and the active site suggested that the ligand cationic aliphatic group could adopt an extended conformation towards Asp132 (Figure 1; **23** pose in purple and Asp132 rotamer in green). The latter residue could adopt another rotameric conformation that reached for the ligand’s cationic group, and both a hydrogen bond and an electrostatic interaction could have occurred (distance cationic nitrogen of the ligand and the anionic oxygen atom of Asp132 2.750 Å). Such an interaction would be consistent with the enzyme inhibition data for hCA IX in which **23** and **24**, compounds with a cationic group, had the lowest ***K*_I_** values. Compound **18** was an analog of **24** without the cationic nitrogen atom with a much higher ***K*_I_** value. Additional analogs such as **16**, **17,** and **19**–**21** similarly had higher ***K*_I_** values compared to **23**.

### 2.4. Molecular Modeling Studies of hCA XII

**Figure 3:**
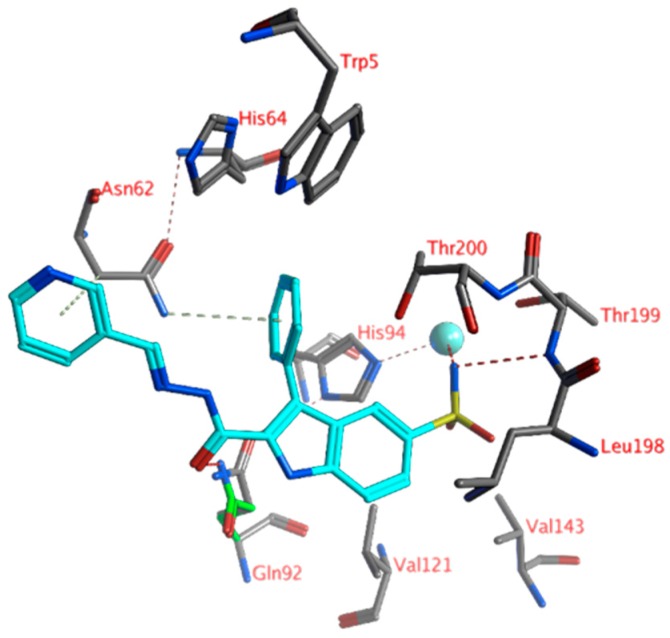
The docked pose of compound **7** (turquoise) in the active site of hCA XII (1jd0). Hydrogen bonds and interactions with the zinc ion are indicated in red dashed lines. H-arene interactions are indicated in yellow dashed lines. The zinc ion is indicated as a turquoise sphere. The Gln92 rotamer that was able to form a hydrogen bond with the ligand is indicated in green sticks.

The lowest ***K*_I_** value for hCA XII was measured for compound **7** (***K*_I_**: 1.4 nM). Again, the sulfonamide moiety formed direct interactions with the Zn^2+^ ion (tetrahedral orientation) and the backbone of Thr199 (Figure 3). The phenyl and the pyridine groups of the ligands formed H-arene interactions with the hydrogen atoms of Asn62. Further hydrophobic interactions were formed with the side chains of His94, Val121, and Leu198. No hydrogen bonds were formed between the ligand and the active site residues. However, the side chain of Gln92 could adopt a conformation in which a hydrogen bond with the ligand carbonyl group was possible (Figure 3).

In general, the docked poses of the compounds, especially the placement of the sulfonamide and the indole parts of the ligands, were similar due to structural similarity between the compounds and the requirements to form an interaction between the sulfonamide and the zinc ion.

**Figure 4:**
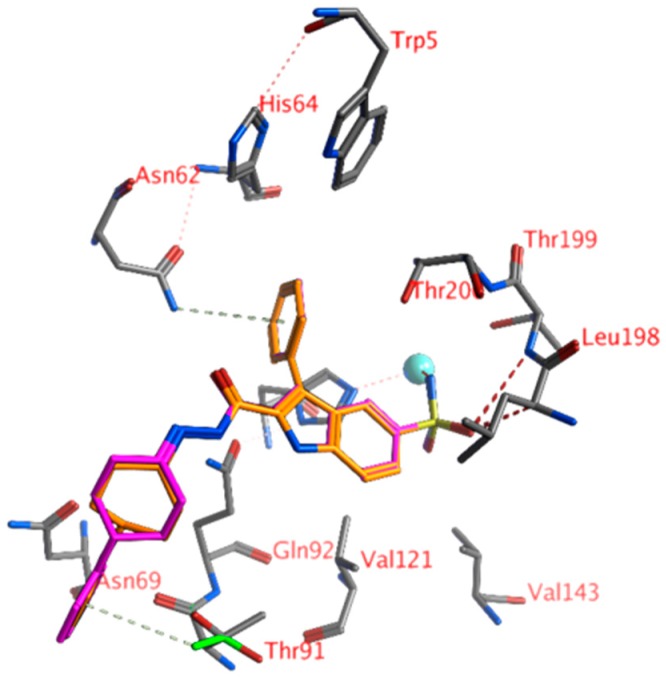
The docked pose of compound **20** (orange) and **21** (purple) in the active site of hCA XII (1jd0). Hydrogen bonds and interactions with the zinc ion are indicated in red dashed lines. H-arene interactions are indicated in yellow dashed lines. The zinc ion is indicated as a turquoise sphere. The Thr91 rotamer that was able to form an H-arene interaction with **21** is indicated in green sticks.

Compounds **18**, **19,** and **20** had bulky hydrophobic groups that could not form hydrophobic interactions with the active site. This may explain the higher ***K*_I_** values for these ligands. Remarkably, compound **21** had a lower ***K*_I_** value even though it also had a bulky hydrophobic substituent. The phenyl group of the ligand may have formed hydrophobic interactions with the side chain of Asn69 and a Thr91 rotamer (with the methyl group pointing to the ligand instead of the hydroxyl group; Figure 4). It seems this interaction was not possible for compounds **18**, **19,** and **20**.

### 2.5. Prediction of ADMET Properties

The potential mutagenicity [23], the reactivity [24] and Lipinski’s parameters (weight, hydrogen bond donor, and acceptor count) of the compounds were calculated using the MOE software package (v2018.0101, Chemical Computing Group Inc., Montreal), and the LogP value [25] was calculated using the ALogPs 2.1 webserver [26]. No violation of Lipinski’s rule of five was found [27,28], indicating that the compounds may have passed cell membranes by passive diffusion. Compounds **7**, **20**, **21,** and **23** were not predicted to be mutagenic, but of these four compounds, only compound **7** was not predicted to be reactive. It should be noted, however, that these were theoretical predictions, and experimental validation is still needed.

## 3. Materials and Methods

Melting points were estimated with a Büchi B-540 (BÜCHI Labortechnik AG, Flawil, Switzerland) melting point apparatus in open capillaries and are presented as uncorrected. Elemental analyses were performed on a Thermo Finnigan Flash EA 1112 (Thermo Electron GmbH, Bremen, Germany) elemental analyzer. IR spectra were recorded on KBr discs using a Perkin-Elmer (Waltham, MA, USA) Model 1600 FT-IR spectrometer. ^1^H NMR, ^13^C-NMR (decoupled), and HSQC-2D spectra were obtained on Varian UNITY INOVA 500, Varian Mercury (Agilent, Palo Alto, CA, USA) 400 MHz FT-NMR spectrophotometers using DMSO-d_6_. The mass spectra were taken on a Waters ZQ micromass LC-MS spectrometer (Waters Corporation, Milford, MA, USA) by using ESI (+) method.

### 3.1. Synthetic Procedures

#### 3.1.1. 2-Benzyl-2-[*N*-(4-sulfonamidophenyl)hydrazono]ethanoate **(1).**

To a solution of 0.01 mole sulfanilamide in 4 mL of 37% HCl, 10 mL of 8% NaNO_2_ aqueous solution was added dropwise at 0 °C. The solution containing the diazonium salt was poured into an ice-cold mixture of 2.3 g (a little excess of 0.01 mole) ethyl 2-benzylacetoacetate, 10 mL of C_2_H_5_OH, 20 mL of H_2_O, and 2.7 g of KOH. The mixture was kept cold overnight. The hydrazone, produced as an oil, was separated, dissolved in (C_2_H_5_)_2_O, washed with H_2_O, and dried over anhydrous Na_2_SO_4_. (C_2_H_5_)_2_O was distilled, and the oily residue was treated with 5 mL of 37% HCl and set aside for 5 h at room temperature. The resulting solid substance was recrystallized from EtOH.

#### 3.1.2. Ethyl 5-(aminosulfonyl)-3-phenyl-1*H*-indole-2-carboxylate **(2).**

A mixture of 0.01 mole ethyl 2-benzyl-2-[N-(4-sulfonamidophenyl)hydrazono]ethanoate and about 10 mL of 37% HCl was heated on a water bath for 4 h, cooled, and poured into 100 mL of H_2_O. The crude product was filtered, washed with H_2_O, and recrystallized from EtOH.

#### 3.1.3. 2-(hydrazinylcarbonyl)-3-phenyl-1*H*-indole-5-sulfonamide **(3).**

For this compound, 3.45 g (0.01 mole) ethyl 5-(aminosulfonyl)-3-phenyl-1*H*-indole-2-carboxylate was dissolved in 20 mL of C_2_H_5_OH, then 4 mL of H_2_NNH_2_-H_2_O was added and refluxed for 6 h, cooled, and kept cold overnight. The resulting crystals were filtered off, washed with (C_2_H_5_)_2_O, and recrystallized from EtOH /DMF.

#### 3.1.4. General procedure for the synthesis of 3-phenyl-5-sulfamoyl-N’-[(heteroaryl)methylidene]-1*H*-indole-2-carbohydrazide/3-phenyl-5-sulfamoyl-N’-[(non)substituedcycloalkylidene/alkylidene]-1*H*-indole-2-carbohydrazide derivatives **(4-24).**

A mixture of 3-phenyl-5-sulfonamidoindole-2-carbohydrazide **(3)** (0.005 mole), an appropriate aldehyde/heteroaromatic aldehyde/ketone/cyclic ketone (0.005 mole), was refluxed in 20 mL absolute ethanol for 6–12 h. The precipitate obtained was purified either by recrystallization from ethanol or by washing with hot ethanol.

#### 3.1.5. 3-Phenyl-2-{[2-(thiophene-2-ylmethylidene)hydrazinyl]carbonyl}-1H-indole-5-sulfonamide **(4)**

Yellow powder (25%); mp 309–310 °C; IR(KBr): υ 3400, 3329, 3250 (NH), 1654 (C=O); 1319, 1147 (S=O); ^1^H NMR (DMSO-d_6_/500 MHz δ (ppm): 7.13 (1H, s, thiophene C_4_-H), 7.20 (2H, s, SO_2_NH_2_), 7.39 (1H, br s, thiophene C_3_-H), 7.44 (1H, br s, thiophene C_5_-H), 7.50-7.57 (5H, m, 3-phenyl C-H), 7.64 (1H, d, J=8.79 Hz, indole C_7_-H), 7.74 (1H, d, J=8.79 Hz, indole C_6_-H), 8.14 (1H, s, indole C_4_-H), 8.31 (1H, s, N=CH), 11.44 (1H, s, CONH), 12.35 (1H, s, indole NH). ^13^C-NMR (HSQC-2D, DMSO-d_6_, 125 MHz) δ (ppm): 113.19 [ind. C_7_ - 7.64 (1H, d, J=8.79 Hz, indole C_7_-H)], 119.06 [ind. C_4_ - 8.14 (1H, s, indole C_4_-H)], 121.79 [ind. C_6_ - 7.74 (1H, d, J=8.79 Hz, indole C_6_-H)], 127.59 [thiophene C_3_ - 7.39 (1H, br s, thiophene C_3_-H),], 128.32 [thiophene C_4_ - 7.13 (1H, s, thiophene C_4_-H)], 129.05, 129.77, 130,16 [3-Phenyl C - 7.50-7.57 (5H, m, 3-phenyl C-H)], 131.88 [thiophene C_5_ - 7.44 (1H, br s, thiophene C_5_-H)], 143.19 [N=CH - 8.31 (1H, s, N=CH)]. Anal. Calcd. for C_20_H_16_N_4_O_3_S_2_ (424.496): C, 56.59; H, 3.80; N, 13.20; S, 15.11. Found: C, 56.27; H, 3.60; N, 11.61; S, 15.43.

#### 3.1.6. 2-({2-[(5-Bromothiophene-2-yl)methylidene]hydrazinyl}carbonyl)-3-phenyl-1H-indole-5-sulfonamide **(5)**

Yellow powder (30%); mp 272–273 °C; IR(KBr): υ 3423, 3300, 3217 (NH), 1654 (C=O); 1334, 1153 (S=O); ^1^H NMR (DMSO-d_6_/500 MHz δ (ppm): 7.20 (2H, s, SO_2_NH_2_), 7.26 (1H, br s, thiophene C_4_-H), 7.31 (1H, br s, thiophene C_3_-H), 7.40 (1H, br s, 3-phenyl C-H), 7.52 (4H, br s, 3-phenyl C-H), 7.64 (1H, d, J=8.78 Hz, indole C_7_-H), 7.73 (1H, dd, J=9.04, 1.46 Hz, indole C_6_-H), 8.13 (1H, s, indole C_4_-H), 8.24 (1H, s, N=CH), 11.52 (1H, s, CONH), 12.32 (1H, s, indole NH). Anal. Calcd. for C_20_H_15_BrN_4_O_3_S_2_ (503.392): C, 47.72; H, 3.00; N, 11.13; S, 12.74. Found: C, 47.82; H, 3.30; N, 11.41; S, 12.90.

#### 3.1.7. 2-({2-[(1-Methyl-1H-pyrrol-2-yl)methylidene]hydrazinyl}carbonyl)-3-phenyl-1H-indole-5-sulfonamide **(6)**

Yellow powder (40%); mp 298–299 °C; IR(KBr): υ 3419, 3313, 3253, 3213 (NH), 1645 (C=O); 1307, 1157 (S=O); ^1^H NMR (DMSO-d_6_/500 MHz δ (ppm): 3.84 (3H, s, pyrrol 1-CH_3_), 6.10 (1H, s, pyrrol C_3_-H), 6.48 (1H, s, pyrrol C_4_-H), 6.98 (1H, s, pyrrol C_5_-H), 7.20 (2H, s, SO_2_NH_2_), 7.41 (2H, d, J=6.83 Hz, 3-phenyl C-H), 7.51-7.56 (3H, m, 3-phenyl C-H), 7.64 (1H, d, J=8.78 Hz, indole C_7_-H), 7.74 (1H, d, J=8.30 Hz, indole C_6_-H), 8.10 (1H, s, indole C_4_-H), 8.13 (1H, s, N=CH), 11.13 (1H, s, CONH), 12.31 (1H, s, indole NH). Anal. Calcd. for C_21_H_19_N_5_O_3_S (421.472): C, 59.84; H, 4.54; N, 16.62; S, 7.61. Found: C, 59.67; H, 4.62; N, 16.81; S, 7.83.

#### 3.1.8. 3-Phenyl-2-{[2-(pyridine-3-ylmethylidene)hydrazinyl]carbonyl}-1H-indole-5-sulfonamide **(7)**

Ivory powder (60%); mp 287.5–287.8 °C; IR(KBr): υ 3360, 3275, 3190 (NH), 1643 (C=O); 1340, 1159 (S=O); ^1^H NMR (DMSO-d_6_/500 MHz δ (ppm): 7.19 (2H, s, SO_2_NH_2_), 7.32-7.53 (6H, m, 3-phenyl C-H and pyridine C_5_-H), 7.65 (1H, d, J=8.78 Hz, indole C_7_-H), 7.74 (1H, d, J=8.30 Hz, indole C_6_-H), 8.14 (2H, br s, indole C_4_-H and N=CH), 8.48 (1H, br s, pyridine C_4_-H), 8.60 (1H, s, pyridine C_6_-H), 8.82 (1H, s, pyridine C_2_-H), 11.64 (1H, s, CONH), 12.38 (1H, s, indole NH). Anal. Calcd. for C_21_H_17_N_5_O_3_S (419.456): C, 60.13; H, 4.09; N, 16.70; S, 7.64. Found: C, 59.80; H, 3.93; N, 16.61; S, 7.43.

#### 3.1.9. 3-Phenyl-2-{[2-(pyridine-4-ylmethylidene)hydrazinyl]carbonyl}-1H-indole-5-sulfonamide **(8)**

Yellow powder (40%); mp 271.5–271.7 °C; IR(KBr): υ 3236, 3178, 3163 (NH), 1666 (C=O); 1321, 1151 (S=O); ^1^H NMR (DMSO-d_6_/500 MHz δ (ppm): 7.21 (2H, s, SO_2_NH_2_), 7.29-7.58 (7H, m, 3-phenyl C-H and pyridine C_3,5_-H), 7.65 (1H, d, J=8.30 Hz, indole C_7_-H), 7.75 (1H, dd, J=8.78, 1.47 Hz, indole C_6_-H), 8.09 (1H, s, indole C_4_-H), 8.15 (1H, br s, N=CH), 8.62 (2H, br s, pyridine C_2,6_-H), 11.75 (1H, s, CONH), 12.38 (1H, s, indole NH). Anal. Calcd. for C_21_H_17_N_5_O_3_S (419.456): C, 60.13; H, 4.09; N, 16.70; S, 7.64. Found: C, 60.33; H, 4.35; N, 16.91; S, 7.93.

#### 3.1.10. 3-Phenyl-2-{[2-(1H-indole-3-ylmethylidene)hydrazinyl]carbonyl}-1H-indole-5-sulfonamide **(9)**

Yellow crystal (20%); mp 264–265 °C; IR(KBr): υ 3373, 3313, 3211 (NH), 1643 (C=O); 1319, 1151 (S=O); ^1^H NMR (DMSO-d_6_/500 MHz δ (ppm): 7.15-7.17 (4H, m, 2.indole C_5,6_-H and SO_2_NH_2_), 7.30-7.45 (2H, m, 3-phenyl C_4_-H and 2.indole C_7_-H), 7.51 (2H, t, J=7.81 Hz, 3-phenyl C_3,5_-H), 7.58 (2H, d, J=7.32 Hz, 3-phenyl C_2,6_-H), 7.64 (1H, d, J=8.78 Hz, indole C_7_-H), 7.70 (1H, dd, J=8.78, 1.46 Hz, indole C_6_-H), 7.79 (1H, d, J=2.93 Hz, 2.indole C_2_-H), 8.13 (1H, s, indole C_4_-H), 8.25 (1H, s, N=CH), 8.26 (1H, d, J=7.81 Hz, 2. indole C_4_-H), 11.07 (1H, s, CONH), 11.59 (1H, s, 2. indole NH), 12.34 (1H, s, indole NH). Anal. Calcd. for C_24_H_19_N_5_O_3_S (457.504): C, 63.01; H, 4.19; N, 15.31; S, 7.01. Found: C, 59.87; H, 4.50; N, 15.61; S, 7.33.

#### 3.1.11. 2-[(2-Methylidenehydrazinyl)carbonyl]-3-phenyl-1H-indole-5-sulfonamide **(10)**

White powder (30%); mp 248.5–248.8 °C; IR(KBr): υ 3244 (NH), 1645 (C=O); 1323, 1149 (S=O); ^1^H NMR (DMSO-d_6_/500 MHz δ (ppm): 7.17 (2H, s, SO_2_NH_2_), 7.35-7.49 (5H, m, 3-phenyl C-H), 7.62 (1H, d, J=8.21 Hz, indole C_7_-H), 7.70 (1H, d, J=8.21 Hz, indole C_6_-H), 8.10 (1H, s, indole C_4_-H), 8.21, 8.24 (2H, 2d, J=3.52 Hz, N=CH_2_), 11.26 (1H, s, CONH), 12.14 (1H, s, indole NH). Anal. Calcd. for C_16_H_14_N_4_O_3_S (342.372): C, 56.13; H, 4.12; N, 16.36; S, 9.37. Found: C, 56.50; H, 3.93; N, 16.60; S, 9.67.

#### 3.1.12. 2-[(2-Ethylidenehydrazinyl)carbonyl]-3-phenyl-1H-indole-5-sulfonamide **(11)**

Ivory powder (27%); mp 250–250.1 °C; IR(KBr): υ 3302, 3221 (NH), 1670 (C=O); 1323, 1145 (S=O); ^1^H NMR (DMSO-d_6_/500 MHz δ (ppm): 1.88 (3H, d, J=3.90 Hz, -CH_3_), 7.18 (2H, s, SO_2_NH_2_), 7.34-7.44 (2H, m, 3-phenyl C_4_-H and N=CH), 7.46-7.54 (4H, m, 3-phenyl C_2,3,5,6_-H), 7.61 (1H, d, J=8.30 Hz, indole C_7_-H), 7.75 (1H, d, J=8.78 Hz, indole C_6_-H), 8.10 (1H, d, J=1.95 Hz, indole C_4_-H), 11.00 (1H, s, CONH), 12.31 (1H, s, indole NH). ^13^CNMR (proton decoupled, DMSO-d_6_, 150 MHz) δ (ppm): 56.15 (-CH_3_), 112.69 (ind. C_7_), 118.64 (ind. C_4_), 119.20 (ind. C_3_), 121.26 (ind. C_6_), 125.46 (ind. C_3a_), 127.17 (3-Phenyl C_4_), 128.65 (3-Phenyl C_3/5_), 129.19 (ind. C_2_), 129.64 (3-Phenyl C_2/6_), 133.03 (ind. C_5_), 136.74 (3-Phenyl C_1_), 139.05 (ind. C_7a_), 148.67 (N=C), 157.75 (CONH). Anal. Calcd. for C_17_H_16_N_4_O_3_S (356.398): C, 57.29; H, 4.52; N, 15.72; S, 9.00. Found: C, 56.96; H, 4.13; N, 16.05; S, 9.37. m/z ESI (positive) 357.36 (M+H)^+^.

#### 3.1.13. 3-Phenyl-2-{[2-(propan-2-ylidene)hydrazinyl]carbonyl}-1H-indole-5-sulfonamide **(12)**

White powder (30%); mp 287.9–288 °C; IR(KBr): υ 3334, 3305, 3217 (NH), 1668 (C=O); 1325, 1147 (S=O); ^1^H NMR (DMSO-d_6_/500 MHz δ (ppm): 1.27 (3H, s, -CH_3_), 1.89 (3H, s, -CH_3_), 7.17 (2H, s, SO_2_NH_2_), 7.45-7.56 (5H, m, 3-phenyl C-H), 7.62 (1H, d, J=8.30 Hz, indole C_7_-H), 7.72 (1H, dd, J=8.30, 1.95 Hz, indole C_6_-H), 7.91 (1H, s, indole C_4_-H), 9.24 (1H, s, CONH), 12.39 (1H, s, indole NH). Anal. Calcd. for C_18_H_18_N_4_O_3_S (370.425): C, 58.36; H, 4.90; N, 15.12; S, 8.66. Found: C, 58.76; H, 5.13; N, 15.05; S, 9.01.

#### 3.1.14. 2-{[2-(Butan-2-ylidene)hydrazinyl]carbonyl}-3-phenyl-1H-indole-5-sulfonamide **(13)**

White powder (78%); mp 285.5–285.6 °C; IR(KBr): υ 3360, 3304, 3224 (NH), 1680 (C=O); 1315, 1151 (S=O); ^1^H NMR (DMSO-d_6_/500 MHz δ (ppm): 0.93-1.04 (3H, m, -CH_3_), 1.26 (3H, s, -CH_3_), 2.20 (2H, d, J=4.88 Hz, -CH_2_-), 7.17 (2H, s, SO_2_NH_2_), 7.45-7.57 (5H, m, 3-phenyl C-H), 7.63 (1H, d, J=8.79 Hz, indole C_7_-H), 7.71 (1H, dd, J=8.79, 1.46 Hz, indole C_6_-H), 7.91 (1H, s, indole C_4_-H), 9.19 (1H, s, CONH), 12.35 (1H, s, indole NH). Anal. Calcd. for C_19_H_20_N_4_O_3_S (384.452): C, 59.36; H, 5.24; N, 14.57; S, 8.34. Found: C, 59.56; H, 5.36; N, 14.15; S, 8.71.

#### 3.1.15. 2-{[2-(Pentan-3-ylidene)hydrazinyl]carbonyl}-3-phenyl-1H-indole-5-sulfonamide **(14)**

White powder (60%); mp 288.2–288.3 °C; IR(KBr): υ 3356, 3300, 3221 (NH), 1680 (C=O); 1319, 1151 (S=O); ^1^H NMR (DMSO-d_6_/500 MHz δ (ppm): 0.63 (3H, s, -CH_3_), 1.00 (3H, s, -CH_3_), 1.60 (2H, d, J=6.04 Hz, -CH_2_-), 2.20 (2H, d, J=6.04 Hz, -CH_2_-), 7.15 (2H, s, SO_2_NH_2_), 7.46-7.58 (5H, m, 3-phenyl C-H), 7.62 (1H, d, J=8.78 Hz, indole C_7_-H), 7.71 (1H, dd, J=8.23, 1.65 Hz, indole C_6_-H), 7.85 (1H, s, indole C_4_-H), 9.20 (1H, s, CONH), 12.39 (1H, s, indole NH). Anal. Calcd. for C_20_H_22_N_4_O_3_S (398.478): C, 60.28; H, 5.56; N, 14.06; S, 8.05. Found: C, 59.96; H, 5.16; N, 14.40; S, 8.36.

#### 3.1.16. 2-{[2-(4-Methylpentan-2-ylidene)hydrazinyl]carbonyl}-3-phenyl-1H-indole-5-sulfonamide **(15)**

White powder (50%); mp 255.1–255.2 °C; IR(KBr): υ 3352, 3321, 3265 (NH), 1674 (C=O); 1332, 1151 (S=O); ^1^H NMR (DMSO-d_6_/500 MHz δ (ppm): 0.83 (6H, d, J=5.49 Hz, -CH(CH_3_)_2_), 1.24 (3H, s, -CH_3_), 1.84-1.88 (1H, m, -CH-), 2.05 (2H, d, J=5.49 Hz, -CH_2_-), 7.16 (2H, s, SO_2_NH_2_), 7.47-7.56 (5H, m, 3-phenyl C-H), 7.62 (1H, d, J=8.78 Hz, indole C_7_-H), 7.72 (1H, dd, J=8.78, 1.65 Hz, indole C_6_-H), 7.91 (1H, s, indole C_4_-H), 9.20 (1H, s, CONH), 12.36 (1H, s, indole NH). Anal. Calcd. for C_21_H_24_N_4_O_3_S (412.505): C, 61.14; H, 5.86; N, 13.58; S, 7.77. Found: C, 60.83; H, 5.50; N, 13.35; S, 7.56.

#### 3.1.17. 2-[(2-Cyclopentylidenehydrazinyl)carbonyl]-3-phenyl-1H-indole-5-sulfonamide **(16)**

White powder (84%); mp 313.1–313.2 °C; IR(KBr): υ 3336, 3234, 3138 (NH), 1658 (C=O); 1344, 1153 (S=O); ^1^H NMR (DMSO-d_6_/500 MHz δ (ppm): 1.50-1.65 (6H, m, -cyclopentyl C-H), 2.30 (2H, m, -cyclopentyl C-H), 7.17 (2H, s, SO_2_NH_2_), 7.50-7.59 (5H, m, 3-phenyl C-H), 7.61 (1H, d, J=8.78 Hz, indole C_7_-H), 7.71 (1H, dd, J=8.78, 0.97 Hz, indole C_6_-H), 7.89 (1H, s, indole C_4_-H), 9.01 (1H, s, CONH), 12.39 (1H, s, indole NH). ^13^CNMR (proton decoupled, DMSO-d_6_, 150 MHz) δ (ppm): 24.08, 24.11 (cyclopentyl C_3/4_), 26.62, 32.59 (cyclopentyl C_2/5_), 112.64 (ind. C_7_), 118.53 (ind. C_4_), 121.35 (ind. C_6_), 126.16 (ind. C_3a_), 127.93 (3-Phenyl C_4_), 128.90 (3-Phenyl C_3/5_), 129.19 (ind. C_2_), 130.19 (3-Phenyl C_2/6_), 132.85 (ind. C_5_), 136.35 (3-Phenyl C_1_), 136.58 (ind. C_7a_), 156.90 (N=C), 167.18 (CONH). Anal. Calcd. for C_20_H_20_N_4_O_3_S (396.462): C, 60.59; H, 5.08; N, 14.13; S, 8.09. Found: C, 60.73; H, 5.10; N, 13.85; S, 7.86. m/z ESI (positive) 396.40 (M+H)^+^.

#### 3.1.18. 2-[(2-Cyclohexylidenehydrazinyl)carbonyl]-3-phenyl-1H-indole-5-sulfonamide **(17)**

White powder (80%); mp 285.5–285.6 °C; IR(KBr): υ 3358, 3296, 3147 (NH), 1676 (C=O); 1317, 1149 (S=O); ^1^H NMR (DMSO-d_6_/500 MHz δ (ppm): 1.34 (2H, s, -cyclohexyl C-H), 1.49 (2H, s, - cyclohexyl C-H), 1.60 (4H, s, -cyclohexyl C-H), 2.28 (2H, s, -cyclohexyl C-H), 7.16 (2H, s, SO_2_NH_2_), 7.45-7.57 (5H, m, 3-phenyl C-H), 7.61 (1H, d, J=8.79 Hz, indole C_7_-H), 7.72 (1H, dd, J=7.32, 1.46 Hz, indole C_6_-H), 7.94 (1H, s, indole C_4_-H), 9.52 (1H, s, CONH), 12.36 (1H, s, indole NH). ^13^CNMR (proton decoupled, DMSO-d_6_, 150 MHz) δ (ppm): 24.80 (cyclohexyl C_4_), 25.50, 26.26 (cyclohexyl C_3/5_), 26.56, 34.69 (cyclohexyl C_2/6_), 112.56 (ind. C_7_), 118.46 (ind. C_4_), 121.18 (ind. C_6_), 125.95 (ind. C_3a_), 127.59(3-Phenyl C_4_), 128.93 (3-Phenyl C_3/5_), 129.40 (ind. C_2_), 130.10 (3-Phenyl C_2/6_), 132.82 (ind. C_5_), 136.29 (3-Phenyl C_1_), 136.64 (ind. C_7a_), 157.26 (N=C), 161.59 (CONH). Anal. Calcd. for C_21_H_22_N_4_O_3_S (410.489): C, 61.44; H, 5.40; N, 13.65; S, 7.81. Found: C, 61.83; H, 5.17; N, 13.86; S, 7.46. m/z ESI (positive) 411.42 (M+H)^+^.

#### 3.1.19. 2-{[2-(4-Methylcyclohexylidene)hydrazinyl]carbonyl}-3-phenyl-1H-indole-5-sulfonamide **(18)**

White powder (75%); mp 295.6–295.7 °C; IR(KBr): υ 3350, 3309, 3165 (NH), 1666 (C=O); 1315, 1147 (S=O); ^1^H NMR (DMSO-d_6_/500 MHz δ (ppm): 0.87 (3H, d, J=5.37 Hz, 4-CH_3_), 1.04-1.14 (1H, s, -cyclohexyl C-H), 1.47-1.58 (4H, m, - cyclohexyl C-H), 1.72-1.80 (2H, m, -cyclohexyl C-H), 2.11-2.22 (1H, m, -cyclohexyl C-H), 2.31 (1H, d, J=12.20 Hz, -cyclohexyl C-H), 7.16 (2H, s, SO_2_NH_2_), 7.42-7.56 (5H, m, 3-phenyl C-H), 7.61 (1H, d, J=8.79 Hz, indole C_7_-H), 7.71 (1H, dd, J=8.79 Hz, indole C_6_-H), 7.93 (1H, s, indole C_4_-H), 9.51 (1H, s, CONH), 12.35 (1H, s, indole NH). Anal. Calcd. for C_22_H_24_N_4_O_3_S (424.515): C, 62.24; H, 5.70; N, 13.20; S, 7.55. Found: C, 61.98; H, 5.63; N, 12.85; S, 7.16.

#### 3.1.20. 2-{[2-(4-Ethylcyclohexylidene)hydrazinyl]carbonyl}-3-phenyl-1H-indole-5-sulfonamide **(19)**

White powder (74%); mp 282.4–282.5 °C; IR(KBr): υ 3348, 3311, 3176 (NH), 1666 (C=O); 1315, 1147 (S=O); ^1^H NMR (DMSO-d_6_/500 MHz δ (ppm): 0.85 (3H, t, J=7.32 Hz, 4-CH_2_CH_3_), 1.16 (1H, t, J=7.32 Hz, -cyclohexyl C-H), 1.21 (2H, t, J=6.83 Hz, 4-CH_2_CH_3_), 1.35 (1H, br s, -cyclohexyl C-H), 1.49 (1H, t, J=13.67 Hz, -cyclohexyl C-H), 1.62 (2H, d, J=12.20 Hz, -cyclohexyl C-H), 1.75 (1H, d, J=13.67 Hz, -cyclohexyl C-H), 1.85 (1H, d, J=9.27 Hz, -cyclohexyl C-H), 2.14 (1H, t, J=11.71 Hz, -cyclohexyl C-H), 2.32 (1H, d, J=12.20 Hz, -cyclohexyl C-H), 7.17 (2H, s, SO_2_NH_2_), 7.37-7.57 (5H, m, 3-phenyl C-H), 7.62 (1H, d, J=8.79 Hz, indole C_7_-H), 7.72 (1H, dd, J=8.79 Hz, indole C_6_-H), 7.93 (1H, s, indole C_4_-H), 9.50 (1H, s, CONH), 12.37 (1H, s, indole NH). Anal. Calcd. for C_23_H_24_N_4_O_3_S (424.515): C, 62.24; H, 5.70; N, 13.20; S, 7.55. Found: C, 62.55; H, 5.38; N, 13.63; S, 7.47.

#### 3.1.21. 2-{[2-(4-Propylcyclohexylidene)hydrazinyl]carbonyl}-3-phenyl-1H-indole-5-sulfonamide **(20)**

White powder (46%); mp 249.7–250 °C; IR(KBr): υ 3354, 3331, 3236 (NH), 1662 (C=O); 1342, 1143 (S=O); ^1^H NMR (DMSO-d_6_/500 MHz δ (ppm): 0.86 (3H, t, J=7.32 Hz, 4-CH_2_CH_2_CH_3_), 1.06 (1H, t, J=7.32 Hz, -cyclohexyl C-H), 1.15 (2H, d, J=6.34 Hz, 4-CH_2_CH_2_CH_3_), 1.28 (2H, q, J=7.32 Hz, 4-CH_2_CH_2_CH_3_), 1.45-1.51 (3H, m, - cyclohexyl C-H), 1.60 (1H, d, J=12.20 Hz, -cyclohexyl C-H), 1.74 (1H, d, J=13.67 Hz, -cyclohexyl C-H), 1.84 (1H, d, J=14.00 Hz, -cyclohexyl C-H), 2.14 (1H, t, J=10.74 Hz, -cyclohexyl C-H), 2.30-2.36 (1H, m, -cyclohexyl C-H), 7.16 (2H, s, SO_2_NH_2_), 7.36-7.57 (5H, m, 3-phenyl C-H), 7.61 (1H, d, J=8.79 Hz, indole C_7_-H), 7.72 (1H, d, J=8.79 Hz, indole C_6_-H), 7.93 (1H, s, indole C_4_-H), 9.50 (1H, s, CONH), 12.36 (1H, s, indole NH). Anal. Calcd. for C_24_H_28_N_4_O_3_S (452.569): C, 63.69; H, 6.24; N, 12.38; S, 7.09. Found: C, 63.45; H, 6.47; N, 12.78; S, 7.49.

#### 3.1.22. 2-{[2-(4-Phenylcyclohexylidene)hydrazinyl]carbonyl}-3-phenyl-1H-indole-5-sulfonamide **(21)**

White powder (80%); mp 312.8–313 °C; IR(KBr): υ 3358, 3319, 3280 (NH), 1670 (C=O); 1338, 1159 (S=O); ^1^H NMR (DMSO-d_6_/500 MHz δ (ppm): 1.28-1.41 (1H, m, -cyclohexyl C-H), 1.58-1.77 (3H, m, -cyclohexyl C-H), 1.87 (1H, d, J=13.18 Hz, -cyclohexyl C-H), 1.96 (1H, br s, -cyclohexyl C-H), 2.26-2.39 (1H, m, -cyclohexyl C-H), 2.46 (1H, d, J=13.18 Hz, -cyclohexyl C-H), 2.78 (1H, m, -cyclohexyl C-H), 7.17 (2H, s, SO_2_NH_2_), 7.19-7.21 (3H, m, cyclohexyl 4-phenyl C-H), 7.30 (2H, t, J=7.81 Hz, cyclohexyl 4-phenyl C-H), 7.42-7.48 (1H, m, 3-phenyl C-H), 7.49-7.55 (4H, m, 3-phenyl C-H), 7.63 (1H, d, J=8.79 Hz, indole C_7_-H), 7.71 (1H, dd, J=8.79, 0.98 Hz, indole C_6_-H), 7.95 (1H, s, indole C_4_-H), 9.61 (1H, s, CONH), 12.37 (1H, s, indole NH). Anal. Calcd. for C_27_H_26_N_4_O_3_S (486.585): C, 66.65; H, 5.39; N, 11.51; S, 6.59. Found: C, 66.38; H, 5.57; N, 11.88; S, 8.94.

#### 3.1.23. 2-{[2-(4-tert-buthylcyclohexylidene)hydrazinyl]carbonyl}-3-phenyl-1H-indole-5-sulfonamide **(22)**

White powder (55%); mp 284.9–285.2 °C; IR(KBr): υ 3358, 3284, 3161 (NH), 1662 (C=O); 1342, 1143 (S=O); ^1^H NMR (DMSO-d_6_/500 MHz δ (ppm): 0.83 (9H, s, 3 x CH_3_), 1.10-1.23 (3H, m, -cyclohexyl C-H), 1.45 (1H, td, J=14.15, 4.88 Hz, -cyclohexyl C-H), 1.64 (1H, d, J=10.74 Hz, -cyclohexyl C-H), 1.80-1.89 (2H, m, -cyclohexyl C-H), 2.13 (1H, t, J=10.74 Hz, -cyclohexyl C-H), 2.36 (1H, d, J=12.69 Hz, -cyclohexyl C-H), 7.16 (2H, s, SO_2_NH_2_), 7.47-7.56 (5H, m, 3-phenyl C-H), 7.61 (1H, d, J=8.79 Hz, indole C_7_-H), 7.71 (1H, d, J=8.79 Hz, indole C_6_-H), 7.94 (1H, s, indole C_4_-H), 9.49 (1H, s, CONH), 12.36 (1H, s, indole NH). Anal. Calcd. for C_25_H_30_N_4_O_3_S (466.595): C, 64.35; H, 6.48; N, 12.01; S, 6.87. Found: C, 64.28; H, 6.35; N, 11.90; S, 6.47.

#### 3.1.24. 2-{[2-(1-Methylpiperidine-4-ylidene)hydrazinyl]carbonyl}-3-phenyl-1H-indole-5-sulfonamide **(23)**

White powder (65%); mp 250.1–250.3 °C; IR(KBr): υ 3352, 3334, 3305 (NH), 1666 (C=O); 1315, 1159 (S=O); ^1^H NMR (DMSO-d_6_/500 MHz δ (ppm): 1.71 (2H, br s, piperidine C_3/5_-H), 2.17 (5H, br s, N-CH_3_ and piperidine C_2/6_ -H), 2.30 (2H, br s, piperidine C_3/5_-H), 2.42 (2H, br s, piperidine C_2/6_ -H), 7.17 (2H, s, SO_2_NH_2_), 7.38-7.54 (5H, m, 3-phenyl C-H), 7.61 (1H, d, J=8.79 Hz, indole C_7_-H), 7.72 (1H, dd, J=8.78, 1.46 Hz, indole C_6_-H), 7.96 (1H, s, indole C_4_-H), 9.66 (1H, s, CONH), 12.39 (1H, s, indole NH). ^13^CNMR (proton decoupled, DMSO-d_6_, 150 MHz) δ (ppm): 26.42, 33.99 (piperidine C_3/5_), 45.20 (N-CH_3_), 53.88, 55.29 (piperidine C_2/6_), 112.66 (ind. C_7_), 118.52 (ind. C_4_), 118.60 (ind. C_3_), 121.28 (ind. C_6_), 125.86 (ind. C_3a_), 127.59 (3-Phenyl C_4_), 128.93 (3-Phenyl C_3/5_), 132.93 (ind. C_5_), 130.19 (3-Phenyl C_2/6_), 136.36 (3-Phenyl C_1_), 136.64 (ind. C_7a_), 157.51 (N=C), 158.57 (CONH). ^13^C-NMR (HSQC-2D, DMSO-d_6_, 125 MHz) δ (ppm): 26.40 [piperidine C_3/5_ - 1.71 (2H, br s, piperidine C_3/5_-H)], 33.95 [piperidine C_3/5_- 2.30 (2H, br s, piperidine C_3/5_-H)], 45.20 [N-CH_3_ - 2.17 (5H, br s, N-CH_3_ and piperidine C_2/6_-H)], 53.87 [piperidine C_2/6_- 2.17 (5H, br s, N-CH_3_ and piperidine C_2/6_ -H)], 55.28 [piperidine C_2/6_—2.42 (2H, br s, piperidine C_2/6_ -H)], 112.63 [ind. C_7_ - 7.61 (1H, d, J=8.79 Hz, indole C_7_-H)], 118.55 [ind C_4_ -7.96 (1H, s, indole C_4_-H)], 121.27 [ind. C_6_ - 7.72 (1H, dd, J=8.78, 1.46 Hz, indole C_6_-H)], 127.58 [3-phenyl C_4_ - 7.38–7.54 (5H, m, 3-phenyl C-H)], 128.92 [3-phenyl C_3/5_ - 7.38–7.54 (5H, m, 3-phenyl C-H)], 130.14 [3-phenyl C_2/6_ - 7.38–7.54 (5H, m, 3-phenyl C-H)]. Anal. Calcd. for C_21_H_23_N_5_O_3_S (425.504): C, 59.28; H, 5.45; N, 16.46; S, 7.54. Found: C, 58.87; H, 5.30; N, 16.20; S, 7.36.

#### 3.1.25. 2-{[2-(1-Benzylpiperidine-4-ylidene)hydrazinyl]carbonyl}-3-phenyl-1H-indole-5-sulfonamide **(24)**

White powder (70%); mp 251.2–251.4 °C; IR(KBr): υ 3379, 3352, 3265 (NH), 1685 (C=O); 1311, 1176 (S=O); ^1^H NMR (DMSO-d_6_/500 MHz δ (ppm): 1.73 (2H, br s, piperidine C-H), 2.26-2.31 (4H, m, piperidine C-H), 2.48 (2H, s, piperidine C-H), 3.37 (2H, s, N-CH2-), 7.16 (2H, s, SO_2_NH_2_), 7.25-7.35 (5H, m, benzyl C-H), 7.39-7.45 (1H, m, 3-phenyl C-H), 7.49-7.55 (4H, m, 3-phenyl C-H), 7.61 (1H, d, J=8.30 Hz, indole C_7_-H), 7.72 (1H, d, J=8.79 Hz, indole C_6_-H), 7.96 (1H, s, indole C_4_-H), 9.67 (1H, s, CONH), 12.37 (1H, s, indole NH). ^13^CNMR (proton decoupled, DMSO-d_6_, 150 MHz) δ (ppm): 26.42, 33.93 (piperidine C_3/5_), 51.50, 52.72 (piperidine C_2/6_), 61.01 (N-CH_2_-Ph), 112.53 (ind. C_7_), 118.45 (ind. C_4_), 122.49 (ind. C_6_), 125.76 (ind. C_3a_), 126. 84, 127.40 (2 x Phenyl C_4_), 128.08, 128.61 (2 x Phenyl C_3/5_), 128.82 (ind. C_2_), 129.38, 130.08 (2 x Phenyl C_2/6_), 132.90 (ind. C_5_), 136.30, 136.57 (2 x Phenyl C_1_), 138.30 (ind. C_7a_), 157.50 (N=C), 158.50 (CONH). Anal. Calcd. for C_27_H_27_N_5_O_3_S (501.599): C, 64.65; H, 5.43; N, 13.96; S, 6.39. Found: C, 64.87; H, 5.20; N,14.22; S, 6.16. m/z ESI (positive) 502.63 (M+H)^+^.

### 3.2. Enzyme Inhibition Studies

A stopped-flow instrument (SX.18 MV-R Applied Photophysics model) was used for assaying the CO_2_ hydration activity of various CA isozymes, as reported by Khalifah [29]. Phenol red (at a concentration of 0.2 mM) was used as indicator, working at the absorbance maximum of 557 nm with 10 mM Hepes (pH 7.4) as a buffer and 0.1 M NaClO_4_ (for constantly maintaining the ionic strength; this anion was not inhibitory in the used concentration) following the CA-catalyzed CO_2_ hydration reaction for a period of 5–10 s. Saturated CO_2_ solutions in water at 25 °C were used as substrates. Stock solutions of inhibitors were prepared at a concentration of 10 mM (in DMSO/water 1:1, *v*/*v*) and dilutions up to 0.01 nM were done with the assay buffer mentioned above. At least seven different inhibitor concentrations were used for measuring the inhibition constant. For allowing the complete formation of the enzyme-inhibitor adduct, the inhibitor and the enzyme were pre-incubated for 15 min. IC_50_ values were obtained from dose response curves working at seven different concentrations of the test compound (from 0.1 nM to 50 mM) by fitting the curves using PRISM (www.graphpad.com) and non-linear least squares methods, the obtained values representing the mean of at least three different determinations. The inhibition constants (***K*_I_**) were derived from the IC_50_ values by using the Cheng-Prusoff equation as follows: ***K*_I_**=IC_50_/(1+[S]/***K*_M_**) where [S] represents the CO_2_ concentration at which the measurement was carried out, and ***K*_M_** represents the concentration of the substrate at which the enzyme activity was at half maximal. As reported earlier, all enzymes used were recombinant and produced in *Escherichia Coli*. [30,31,32,33]. The concentrations of enzymes used in the assay were: hCA I, 11.9 nM; hCA II, 7.7 nM; hCA IX, 9.1 nM, and hCA XII, 11.5 nM.

### 3.3. Molecular Modeling Studies

The three-dimensional structures of all ligands were prepared in their lowest energy conformation using the MOE software package (v2018.0101, Chemical Computing Group, Inc, Montreal, Canada). The sulfonamide nitrogen atoms were assigned a negative charge (R-SO_2_NH-), and the ligands were energy minimized (MMFF94x force field).

All protein structures were obtained from the RCSB protein databank: hCA I in complex with topiramate (pdb: 3lxe, 1.90 Å), hCA II in complex with water (pdb: 4e3d, 1.60 Å), hCA IX in complex with acetazolamide (pdb: 3iai; 2.20 Å), and hCA XII in complex with acetazolamide (pdb: 1jd0; 1.50 Å). The protein atoms and the active site zinc ions were retained, and all other atoms were omitted. The remaining structure was protonated using the protonate 3D functionality of MOE, and subsequently, the obtained structure was energy-minimized (AMBER14:EHT) [34]. Finally, the obtained protein models were superposed on the hCA I structure using the backbone Cα-atoms and all Zn^2+-^ions, zinc-binding histidines, and the overall backbone atoms superposed well [root-mean-square deviation (RMSD) value: 1.281 Å].

Docking calculations were performed using the FlexX docking tool (v2.3.2; BioSolveIT GmbH, St. Augustin, Germany) within MOE. The binding pocket was defined as all residues within 6.5 Å of the reference ligand, acetazolamide. The sulfonamide tail of the ligands was forced to adopt a similar orientation and interactions to the Zn^2+-^ion as observed for acetazolamide using a pharmacophore model. All ligands were docked fifty times, and the best scoring three poses were subjected to refinement calculations. To this end, the ligand and the binding pocket residues were energy minimized and rescored using a GBVI/WSA force field [35].

## 4. Conclusions

A total of 21 new indole-based sulfonamide derivatives were synthesized and tested against the tumor-associated hCA IX and XII and the widespread off-targets, hCA I and II. The compounds showed potent inhibition in the lower nanomolar range and at least ~six-fold selectivity for hCA IX and XII over hCA I and II. Molecular modeling studies were subsequently performed to gather insight in the possible binding modes between the newly developed CAIs and their respective target hCAs. This information may result in more potent and more selective CAIs for hCA IX and XII.

## Figures and Tables

**Table 1:** Inhibition data of hCA I, II, IX, XII with compounds **4-24** and the standard inhibitor acetazolamide **(AAZ)** by a stopped flow CO_2_ hydrase assay.

			*K*_I_^*^ (nM)			
Cpd.	R	R_1_	hCA I	hCA II	hCAIX	hCA XII
**4**	H	Thiophene-2-yl	6667.5	>10,000	47.4	4.5
**5**	H	5-Br-thiophene-2-yl	9408.7	>10,000	48.4	75.1
**6**	H	1-CH_3_-pyrrol-2-yl	9572	>10,000	44.3	64.4
**7**	H	pyridine-3-yl	8592.1	8906.7	35.8	1.4
**8**	H	pyridine-4-yl	3134.9	3487.4	15.8	6.6
**9**	H	indole-3-yl	>10,000	>10,000	206.8	77.3
**10**	H	H	7479.2	9369.7	32.9	23.8
**11**	H	CH_3_	4056.8	5866.8	12.9	62.6
**12**	CH_3_	CH_3_	9558.6	8498.4	47.9	87.9
**13**	CH_3_	C_2_H_5_	9619.6	8029.7	42.1	92.4
**14**	C_2_H_5_	C_2_H_5_	9246.3	6621.8	43.7	9.4
**15**	CH_3_	isobuthyl	8652.3	6562.3	187.8	27.9
**16**	cyclopentyl		>10,000	5960.5	476.1	97.2
**17**	cyclohexyl		>10,000	5867.9	191	86.2
**18**	4-methylcyclohexyl		>10,000	8942.5	418.9	139.1
**19**	4-ethylcyclohexyl		>10,000	1282.7	475.3	219.7
**20**	4-propylcyclohexyl		>10,000	795.8	347.7	153.6
**21**	4-phenylcyclohexyl		>10,000	830.7	32.3	14.8
**22**	4-*ter*t-buthylcyclohexyl		>10,000	3380.8	17.4	26.6
**23**	1-methylpiperidin-4-yl		>10,000	309	9.2	62.9
**24**	1-benzylpiperidine-4-yl		7384.7	372.4	11.7	9.4
**AAZ**	-	-	250	12.5	25	5.7

* Mean from three different assays by a stopped flow technique (errors were in the range of 5–10 % of the reported values). hCA= human carbonic anhydrases, AAZ= acetazolamide.

## Abbreviations

CA	Carbonic Anhydrases
hCA	Human Carbonic Anhydrases
CAI	Carbonic Anhydrase Inhibitor
